# Medicinal Plants Used in the Management of Sexual Dysfunction, Infertility and Improving Virility in the East African Community: A Systematic Review

**DOI:** 10.1155/2023/6878852

**Published:** 2023-08-12

**Authors:** Christine Kyarimpa, Christine Betty Nagawa, Timothy Omara, Silver Odongo, Patrick Ssebugere, Solomon Omwoma Lugasi, Ivan Gumula

**Affiliations:** ^1^Department of Chemistry, Faculty of Science, Kyambogo University, P.O. Box 1, Kampala, Uganda; ^2^Department of Forestry, Biodiversity and Tourism, College of Agricultural and Environmental Sciences, Makerere University, P.O. Box 7062, Kampala, Uganda; ^3^Chemistry Division (Food Safety Laboratories), Testing Department, Standards Directorate, Uganda National Bureau of Standards, P.O. Box 6329, Kampala, Uganda; ^4^Department of Chemistry, College of Natural Sciences, Makerere University, P.O. Box 7062, Kampala, Uganda; ^5^Department of Physical Sciences, Jaramogi Oginga Odinga University of Science and Technology, P.O. Box 210, Bondo 40601, Kenya

## Abstract

Sexual disorders such as erectile dysfunction (ED), sterility, and sexual inappetence represent some of the complex reproductive challenges that require addressing the underlying causes. The aim of this paper was to systematically synthesize literature on the ethnobotany, phytochemistry, bioactivities, and safety of plants used as remedies for managing sexual dysfunction and infertility, and improving fertility and virility in the EAC. Through an extensive review conducted in multidisciplinary electronic databases, 171 plant species were identified to have been reported for the management of sexual inappetence (i.e., used as aphrodisiacs, 39.4%), ED (35.9%), infertility (18.7%), and increasing fertility (6.0%). The most used plants are *Mondia whitei*, *Acalypha villicaulis*, *Combretum illairii*, *Erythrina abyssinica*, *Pappea capensis*, *Rhus vulgaris*, and *Warburgia ugandensis* while roots (44.9%), leaves (21.8%), stem and root barks (16.7%) of shrubs (35%), trees (31%), herbs (26%), and climbers (8%) are the preferred organs for making decoctions (69%). The research strides to date indicate that *Citropsis articulata*, *Cola acuminata*, *Ekebergia capensis*, *Plumbago zeylanica*, *Tarenna graveolens*, *Urtica massaica*, and *Zingiber officinale* have been assessed for their bioactivity. The majority (71.4%) of the plants either increased testosterone levels and mounting frequency or elicited prosexual stimulatory effects in male rats. More studies investigating the relevant pharmacological activities (aphrodisiac, fertility, and phosphodiesterase-5 inhibitory activities), safety aspects, responsible compounds, and clinical studies are warranted to establish the pharmacological potential of the unstudied species and elucidate the mechanism of action of the bioactive compounds.

## 1. Introduction

One of the universal interests enshrined in sustainable development goal (SDG) 3 is good health and well-being. It is linked to and affects other global goals such as SDG 1 (poverty reduction), SDG 2 (end poverty), and SDG 4 (quality and equitable education) [1]. Critical analysis of the global disease burden shows that one-third of the total world population has more than five ailments [2]. Accordingly, three in five of the global deaths are ascribed to at least one of the four main noncommunicable diseases (NCDs), namely, cancer, diabetes, cardiovascular, and chronic lung diseases [3–5]. Most global mortalities (up to 71%) are due to NCDs [6], and 77% of these occur in low- and middle-income countries due to limited access to medical services and poverty [7]. Whereas the global focus is on the major NCDs, conditions such as sexual dysfunction, infertility, and anaphrodisia (sexual inappetence) represent some of the complex health challenges.

Sexual dysfunction refers to the inability to achieve a normal sexual intercourse. It includes orgasmic disorder, retrograded, retarded, premature ejaculation, and erectile dysfunction [8]. Male erectile dysfunction (ED) or impotence is the inability to achieve or maintain an erection sufficient for satisfactory sexual performance and vaginal intercourse, typically for a period of more than six months [9]. Though to different degrees, ED affects more than 52% of men in the age bracket of 40 and 70 years. Erectile dysfunction is linked with conditions such as diabetes, sedentary lifestyle, hypertension, obesity, hypercholesterolemia, and smoking [10–12].

On the other hand, infertility is a medical condition characterized by failure to establish a clinical pregnancy after one year of regular and unprotected sex [13]. Infertility affects more than 48 million couples worldwide. It can be from either one or both partners, but 50% of all cases are due to male infertility [14]. In women, it may be due to endometriosis (premature ovarian failure) and uterine disorders such as fibroids or thyroid diseases. In males, infertility is associated with defective sperm function, azoospermia, low sperm counts, varicocele, undescended testes, testicular cancer, and low testosterone levels [13, 14]. Other risk factors for infertility include diabetes, sexually transmitted diseases, stress, obesity, drug abuse, age, exposure to environmental toxins, radiotherapy, and other cancer treatments [15, 16].

Sexual inappetence is a common reproductive challenge that accompanies or is a direct consequence of ED and infertility [17, 18]. Sexual inappetence (anaphrodisia or lack of desire/libido) is one of the most common sexual dysfunctions of women. Together, ED, infertility, and sexual inappetence are among the relatively common fecundity challenges that affect couples medically, sexually, and psychologically [17, 18]. With medical advancements in assisted reproduction technologies, the use of synthetic agents such as phosphodiesterase type 5 inhibitors (in intracavernosal injection therapy for ED) and stem cell therapy (for infertility) has been encouraged [13]. However, limited access to medical services, long-term treatment tenure, and side effects of injectable fertility drugs have limited their acceptability among the general population [19–21].

For indigenous communities in developing countries, the use of natural products for prevention and the management of reproductive diseases and conditions are common. The East African Community (EAC) is one of the regions with distinguished ethnomedicinal knowledge and use of natural products [22–25]. The high reliance of these communities on herbal medicine is explained by the exceptionally rich cultural heritage, acceptance, availability, and perceived efficacy [26–28]. In this context, traditional medicine practitioners correlate sicknesses and other medical conditions with their possible causes [25]. For this reason, herbal medications and posology are prescribed based on the supposed cause of the diseases. Critical cases, or those due to supernatural forces, are managed through diviners' interventions [26, 29]. Illnesses are thought to be induced by external polluting influences (e.g., consumption of tabooed foods [30], breaching of taboos, witchcraft-related rites, fetishes or social rules, and use of objects planted by ill people) that interfere with body physiology [26, 31–33]. Therefore, traditional management of diseases involves health practices, knowledge, and beliefs that utilize plants and animal- and mineral-based remedies, dispensing of ritually protective herbal medicines or performing rituals for placating spirits [26, 33]. These perceptions are similar to traditional medicine concepts in other parts of Africa [34].

In the EAC, chronic poverty and resource-constrained healthcare systems are common, and the use of herbal remedies for the treatment of sexual dysfunction (ED) and infertility, and enhancing fertility and virility has been sporadically mentioned in ethnobotanical studies. However, no study has systematically collated literature on these medicinal plants with in-depth description and analysis of their claimed efficacy, phytochemistry, and safety. The aim of this paper was, therefore, to systematically synthesize literature on ethnobotany, phytochemistry, bioactivities, and the safety profile of plants used as remedies for managing sexual dysfunction and infertility, and improving fertility and virility in the EAC. As part of an ongoing project, we aimed at identifying highly cited but unstudied species that could be assessed for their aphrodisiac, fertility and phosphodiesterase-5 inhibitory activities, bioactive phytochemicals, and toxicity profiles. This could open lead to the discovery of molecules that can be used in modern medicine.

## 2. Methods

### 2.1. Study Design, Literature Sources, and Systematic Search Procedures

The Preferred Reporting Items for the Systematic Reviews and Meta-Analyses (PRISMA) 2020 guidelines [35] were followed (Supplementary file 1). The protocol used was registered with the International Prospective Register of Systematic Reviews (PROSPERO) with registration number CRD42022373152 (https://www.crd.york.ac.uk/prospero/display_record.php?ID=CRD42022373152). Nine multidisciplinary electronic databases (Scopus, Web of Science, PubMed, Science Direct, Google Scholar, Wiley Online Library, Taylor and Francis Online, Springer Link, and Scientific Electronic Library Online) and regional university repositories were searched to gather relevant records on ethnobotany, phytochemistry, biological activities, and toxicity of medicinal plants exploited for the management of sexual dysfunction and infertility, and improving fertility and virility in the EAC. The dates on which we last consulted the databases were 7th January 2023, 31st December 2022, 20th November 2022, 20th January 2023, 4th January 2023, 17th January 2023, 11th November 2022, 10th January 2023, 24th November 2022, 2nd December 2022, and 2nd January 2023, respectively.

The EAC was considered as the region encompassing Uganda, Kenya, Tanzania, Rwanda, Burundi, South Sudan, and Democratic Republic of Congo (DRC) from April 2022 [36]. The searches were performed in parallel using search strings specified for a comprehensive search that covered all fields in records but broadened the scope in PubMed advanced search. Within each axis, keywords were combined with the “OR” operator in the Boolean operator and then linked the two axes' search techniques to the “AND” operator. The keywords used were “plant” “erectile dysfunction” “aphrodisiac” “infertility” OR “fertility” “virility” AND “Uganda” “Kenya” “Tanzania” “Rwanda” “Burundi” “South Sudan” “Democratic Republic of Congo.” For example, in Scopus, the search string used was ALL (plants, AND erectile AND dysfunction, AND aphrodisiac, AND uganda) AND (LIMIT-TO (AFFILCOUNTRY, “Uganda”)) OR ALL (plants, AND erectile AND dysfunction, AND aphrodisiac, AND Kenya) AND (LIMIT-TO (AFFILCOUNTRY, “Kenya”)) Kenya OR ALL (plants, AND erectile AND dysfunction, AND aphrodisiac, AND rwanda) AND (LIMIT-TO (AFFILCOUNTRY, “Rwanda”)) OR ALL (plants, AND erectile AND dysfunction, AND aphrodisiac, AND burundi) AND (LIMIT-TO (AFFILCOUNTRY, “Burundi”)) OR ALL (plants, AND erectile AND dysfunction, AND aphrodisiac, AND south Sudan) AND (LIMIT-TO (AFFILCOUNTRY, “South Sudan”)) OR ALL (plants, AND erectile AND dysfunction, AND aphrodisiac, AND Democratic Republic of Congo) AND (LIMIT-TO (AFFILCOUNTRY, “Democratic Republic of Congo”)) OR ALL (plants, AND erectile AND dysfunction, AND aphrodisiac, AND Tanzania) AND (LIMIT-TO (AFFILCOUNTRY,“Tanzania”)) OR ALL (plant, AND infertility AND fertility AND uganda) AND (LIMIT-TO (AFFILCOUNTRY, “Uganda”)) OR ALL (plant, AND infertility AND fertility AND Kenya) AND (LIMIT-TO (AFFILCOUNTRY, “Kenya”)) OR ALL (plant, AND infertility AND fertility AND tanzania) AND (LIMIT-TO (AFFILCOUNTRY, “Tanzania”)) OR ALL (plant, AND infertility AND fertility AND rwanda) AND (LIMIT-TO (AFFILCOUNTRY, “Rwanda”)) OR ALL (plant, AND infertility AND fertility AND burundi) AND (LIMIT-TO (AFFILCOUNTRY, “Burundi”)) OR ALL (plant, AND infertility AND fertility AND south Sudan) AND (LIMIT-TO (AFFILCOUNTRY, “South Sudan”)) OR ALL (plant, AND infertility AND fertility AND democratic republic of congo) AND (LIMIT-TO (AFFILCOUNTRY, “Democratic Republic of Congo”)).

In addition, reference lists of the retrieved studies were also manually searched to access additional articles which were screened for their eligibility for inclusion in the study. The literature search was performed between 1st June 2022 and 20th January 2023.

### 2.2. Study Selection

All search results were imported into EndNote® X9 (Thomson Reuters, Philadelphia, PA, USA), and duplicate reports were removed. The screening was done according to the title and abstract of the articles. This was conducted independently by 4 authors (CK, CBN, TO, and SO). Two independent reviewers (TO and SO) screened the articles against inclusion criteria, and possible contradictions during article selection and/or extraction were obviated through discussions and consensus.

### 2.3. Inclusion and Exclusion Criteria

To refrain the authors from bias, (1) only full-text articles or reports published in or translated into English and French; (2) cross-sectional original papers or reports on the ethnobotany, phytochemistry, bioactivities, and clinical trials of plants used in the management of sexual dysfunction and infertility, and improving fertility and virility in EAC; and (3) reports published online until 20th January 2023 were included. Excluded studies were those that (1) provided no data; (2) were neither from EAC nor full-text articles; (3) reported on the use of plants for managing conditions such as menorrhagia, blocked fallopian tubes, inducing twin birth or birth to a particular sex of children; (4) narrative and systematic reviews, or reports not based on original data (expert opinions, editorials, and perspective papers).

### 2.4. Risk of Bias Assessment

Quality of the considered reports (risk of bias) was established following the Joanna Briggs Institute quality assessment tool [37]. Two authors (TO and SO) independently assessed the quality of the included studies. Variations in the final risk of bias assessment among them were declared by discussing the prespecified criteria. The evaluation tool consisted of seven parameters: (1) appropriate sampling design; (2) correct sampling technique; (3) acceptable sample size; (4) adequate study subject and location explanation; (5) appropriate data investigation; (6) use of valid methods for identification of plants and the conditions that they treat; and (7) use of appropriate statistical/ethnobotanical analysis indices. Because most studies met parameters 5 to 7 that were similar across them, we relied on parameters 1 to 4 to ascertain the risk of bias status. A study that did not meet each parameter was scored as 1 if not 0. The risks for biases were classified as either low (total score, 0-1), moderate (total score, 2), or high (total score, 3-4) [38].

### 2.5. Data Extraction

Data were collated in a predesigned Microsoft Excel 2019 standardized sheet. Information on the reported medicinal plants, such as botanical names (and synonyms), plant family, traditional name(s), growth habit, part(s), and their uses (conditions treated), mode of preparation and administration, isolated pure compounds, and relevant efficacy reports were extracted. For each dataset, the first author's last name, year of publication, and country were also extracted. Missing information in some reports such as local names, growth forms, and misspelled botanical names was checked from Google and botanical databases (WFO Plant List, International Plant Names Index, and Tropicos).

### 2.6. Data Analysis

Descriptive statistical methods were used to analyze the collected data. Results were expressed as ranges, percentages, and frequencies and presented as tables and charts. These analyses were performed in Microsoft Excel 2019 for Windows (Microsoft Corporation, Washington, DC, USA).

## 3. Results and Discussion

### 3.1. Description of Included Studies

**Figure 1:**
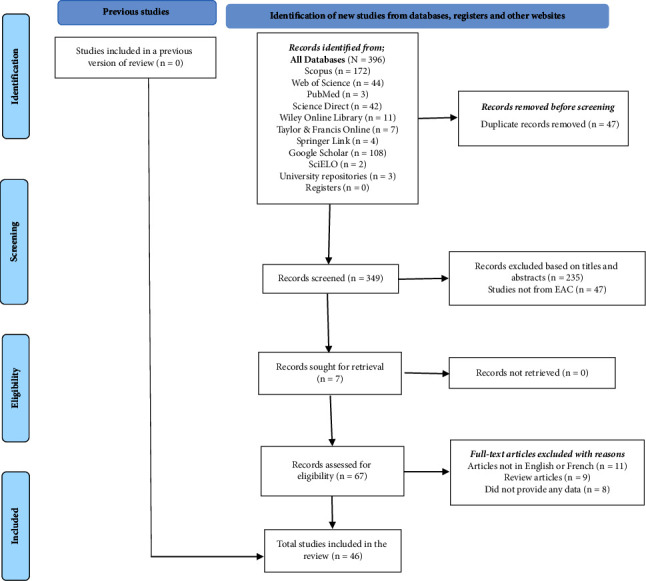
PRISMA flow diagram showing the retrieval and exclusion steps of the systematic review adapted from Page et al. [35].

Our systematic search (Figure 1) retrieved no previous review on the subject in East Africa. From databases, registers, and other websites, the search returned 396 unique reports published between 1962 and 2022. The highest number of reports were from Scopus (*n* = 172) and Google Scholar (*n* = 108), followed by Web of Science (*n* = 44), Science Direct (*n* = 42), Wiley Online Library (*n* = 11), Taylor & Francis Online (*n* = 7), Springer Link (*n* = 4), PubMed (*n* = 3), regional university repositories (*n* = 3), and SciELO (*n* = 2). Of these, duplicates (*n* = 47) were removed, and 349 unique articles were screened. A total of 235 articles were excluded after reading their titles and abstracts, while 47 others were excluded because they were not from any country within the EAC. Therefore, 67 records were assessed for their eligibility and inclusion in the study. Based on the inclusion and exclusion criteria, some full-text articles were excluded with reasons, namely, (i) articles not in English or French (*n* = 11), (ii) review articles (*n* = 9), and (iii) those that did not provide any data (*n* = 8). A manual search resulted in 7 eligible articles. Thus, data were extracted from a total of 46 articles in this systematic review. Regarding the assessment of the risk of bias among studies, most reports were judged as having a low (47.8%) or moderate risk of bias (34.8%) (Supplementary file 2).

### 3.2. Inventory of Medicinal Plants Reported

This review identified 46 reports on plants used in the management and treatment of ED and infertility, and increasing fertility and sexual appetence (virility) in the EAC. Some of the sexual dysfunctions captured from herbalists include erectile disorders, pain during penetration, premature ejaculation, lack of sexual arousal, and short-lasting erections (among men) and lack of orgasm, dyspareunia, lack of sexual arousal, atrophic vaginitis, and short orgasms among women [39].

In total, 171 plant species from 59 botanical families have been reported for the management of sexual inappetence, i.e., used as aphrodisiacs (39.4%), ED (35.9%), infertility (18.7%), and increasing fertility (6.0%) (Table 1). The highest number of plants cited was from Kenya (96), followed by Uganda (66), Tanzania (24), Rwanda (1), and DRC (1). Burundi and South Sudan had no reports on plants in the category under scrutiny. It is not surprising that Kenya ranked the highest, as it is known to have diversified flora with over 7,000 plant species [23, 40]. This is also supported by the fact that most of the ethnobotanical reports reviewed (*n* = 25) were from Kenya as compared to Uganda (*n* = 18), Tanzania (*n* = 7), Rwanda (*n* = 1), and DRC (*n* = 1).

Analysis of transregional distribution of the plants revealed that Uganda and Kenya shared 8 species and Tanzania and Kenya shared 6 species while Kenya and Rwanda shared one species (*Tagetes minuta* L.). Only one plant (*Pachycarpus robusta*) was cited to be used in Uganda, Kenya, and Tanzania [41]. The rest of the countries did not share any plant. Such marked divergence in the use of plants across the region could be due to their preference which is related to specific cultural beliefs and traditions or centred around human relationships [42–44].

**Figure 2:**
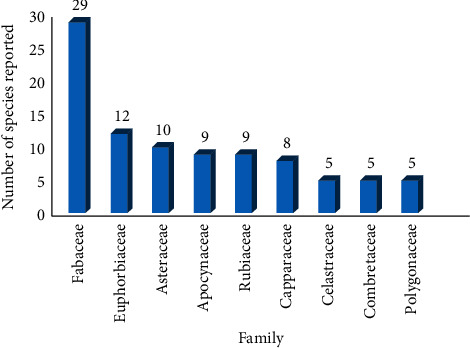
Major botanical families from which remedies used for treating sexual dysfunction and infertility and improving virility are obtained in the EAC.

The majority of the plants retrieved in this study were from families: Fabaceae (16.9%, 29 species), Euphorbiaceae (7.0%, 12 species), Asteraceae (5.8%, 10 species), Apocynaceae, Rubiaceae (5.3%, 9 species each), and Capparaceae (4.7%, 8 species) (Figure 2). Species from these botanical families have been reported to have aphrodisiac and fertility potential in ethnobotanical surveys from Ethiopia [45], Southern Africa [46], Iran [47], and India [48]. The dominance of families, especially Fabaceae and Asteraceae, is due to the extensive range of their distribution across global biomes [49]. Moreover, they contain phytochemicals such as phenolics, tannins, and alkaloids which are known to have therapeutic effects [50, 51].

At the genus level, the most represented genera were *Acacia* (6 species), *Combretum* (5 species), *Cassia* and *Tragia* (3 species each), *Abrus*, *Allium*, *Boscia*, *Cadaba*, *Cleome*, *Croton*, *Impatiens*, *Maytenus*, *Sonchus*, *Uvaria*, *Vachellia*, and *Vernonia* (2 species each). The commonly mentioned plants were *Mondia whitei* (12 times), *Warburgia ugandensis* (4 times), *Acalypha villicaulis*, *Combretum illairii*, *Erythrina abyssinica*, *Pappea capensis*, and *Rhus vulgaris* (3 times each). Some of the plants listed such as *Abrus precatorius*, *Allium sativum*, *Cola acuminata*, *Combretum hereroense*, *Mondia whitei*, *Plumbago zeylanica*, *Ricinus communis*, and *Syzygium guineense* are traditionally used for treating infertility and ED in South Africa [52], Ghana [53], Cameroon, Guinea, Gabon [54], Iran [47], Benin [55], and Ethiopia [45]. It is worth mentioning that organs of some of the highly cited species such as *Abrus precatorius* and *Erythrina abyssinica* are used in Uganda for rituals and ceremonies of love, weddings, and childbirth [56].

In regards to the treatment of infertility, most plan species recorded were indicated to be used for the treatment of female infertility (Table 2). The most cited species were *Erythrina abyssinica* and *Combretum illairii* (3 times each). Interestingly, some species (*Cadaba glandulosa*, *Cadaba farinose*, *Combretum illairii*, *Hoslundia opposita*, and *Allophylus pervilleria*) were shown to be used for the treatment of both female and male infertility, which could make them good candidates for further studies of their biological activities.

### 3.3. Growth Habit, Organs Used, Dosage Forms, and Posology of the Herbal Remedies

**Figure 3:**
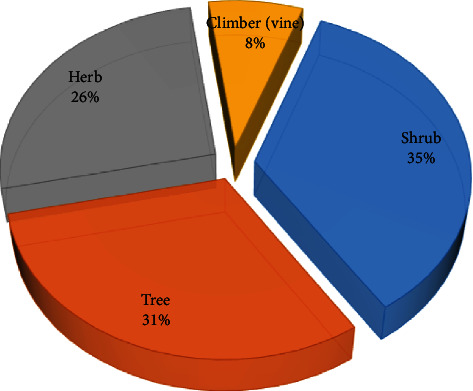
Life form of plants used for the preparation of remedies used in the treatment of erectile dysfunction and infertility, and increasing fertility and virility in the EAC.

**Figure 4:**
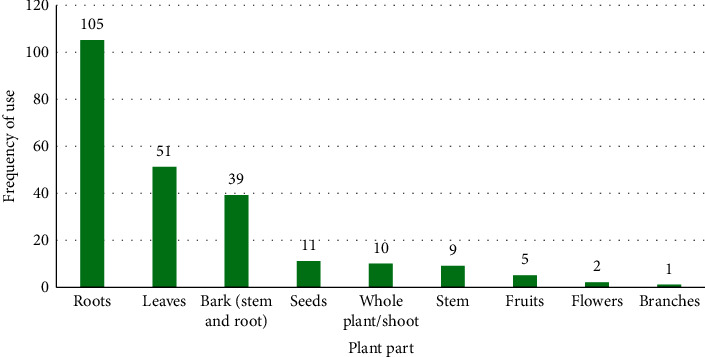
Plant organs used in herbal preparations for treating erectile dysfunction and infertility, and enhancing fertility and virility in the EAC.

The plants occurred as shrubs (35%), trees (31%), herbs (26%), and climbers (8%) (Figure 3).  Figure 4 illustrates which plant organs are widely used in preparation of the herbal remedies, that is, roots (44.9%), leaves (21.8%), and stem and root barks (16.7%). The frequent use of roots is unsustainable but may be linked to the fact that the conditions treated are internal to the body (are hidden), just as root structures are hidden in the ground. On the other hand, the relatively frequent use of leaves could be related to their availability and the fact that they are the photosynthetic sheet of plants that accumulate therapeutic phytochemicals [57].

This review noted a tendency of including more than one plant part and adjuvants in herbal remedies. For multiple plant parts, a total of 13 species were encountered to be used in combination with others. For example, in Kenya, decoction of *Uvaria leptocladon*, *Boscia coriaceae*, and *Combretum hereroense* roots is used for treating ED. For infertility in women, the roots are used with *Croton dichagamus* roots [58, 59]. Similarly, the decoction of *Markhamia zanzibarica* roots mixed with *Uvaria acuminata* roots is administered as an aphrodisiac. For infertility in women, it is used with *Salvadora persica* and *Uvaria acuminata* roots [58, 59]. A striking example of using adjuvants is from Tanzania where roots of *Polygala aphrodisiaca* are cooked with a young cock while *Duosperma kilimandscharicum* leaf and root decoction are taken with goat blood or goat meat soup as an aphrodisiac [41]. The use of cow and goat milk for preparation of *Morus mesozygia* roots as an aphrodisiac was also documented in Kenya [60]. In Uganda, *Acanthus pubescens* leaves are taken in *tonto*, a traditional beer prepared from *Musa* × *paradisiaca* L. var. sapientum fruits [61]. The use of more than one plant organ and adjuvants as witnessed in this review are tailored to various reasons. For instance, it may be an obvious way of masking the toxicity of herbal remedies or hiding the secrecy of the formularies [62, 63].

**Figure 5:**
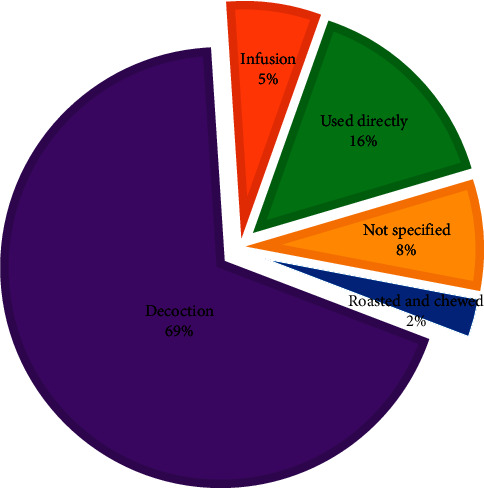
Methods of preparation of herbal remedies used in the treatment of erectile dysfunction and infertility, and enhancing fertility and virility in the EAC.

The commonest method of preparing herbal remedies is decoctions (69%). This could be because decoction procedures allow for better extraction of the bioactive phytochemicals in plant matrices [64]. However, the plant organs may also be used directly, i.e., chewed raw (16%) or prepared as an infusion (5%) and taken (Figure 5). The remedies are administered orally, either by taking decoctions, infusions, and eating or chewing. Only one study reported inhalation of fumes from *Cannabis sativa* leaves for treatment of ED in Uganda [65]. *Aloe volkensii* (leaf decoction) in Kenya when utilized for treating infertility is used as a wash for genitals [58], hinting that internally mediated fertility effects would be unlikely when such herbal remedies are administered orally. While most of the plants had their method of preparation and routes of administration indicated in the use reports, up to 8% of the species identified did not have specifications of the method of preparation and administration of the herbal remedies.

### 3.4. Bioactivity and Phytochemistry of the Reported Plants

To decipher the therapeutic mechanisms and compounds responsible for the bioactivities of the plants reported in EAC, a holistic review of their bioactivity related to the traditional claims and phytochemistry was undertaken. However, only five reports on bioactivity from EAC were encountered for seven plants reported in this study. In this context, the aqueous extract of *Citropsis articulata* root bark was reported to increase the *in vivo* levels of serum testosterone and mounting frequency in male rats [66, 67]. Joseph et al. [68] found that aqueous extract of *Cola acuminata* (fruits) and *Zingiber officinale* (rhizome) had no significant effect on mounting frequency and testosterone levels in rats. Aqueous extract of *Tarenna graveolens* roots increased testosterone levels but had no significant effect on mounting frequency while aqueous extract of *Urtica massaica* leaves elicited no appreciable increase in mounting frequency and testosterone levels in male rats [68]. Other reports were for ethanolic stem bark extract of *Ekebergia capensis* which alleviated sexual dysfunction by increasing the mounting frequency and testosterone levels of male rats to 2.38 ± 0.02 ng/ml, 7.68 ± 0.66, and 14.5 ± 0.777 ng/mL at doses of 300, 400, and 500 mg/kg, respectively [69]. The latest report is on *Plumbago zeylanica*, whose aqueous root extract administered at 150, 300, and 450 mg/kg was found to elicit prosexual stimulatory effects in male rats [70]. Though some of these reports supported the traditional use of the medicinal plants, most studies performed preliminary phytochemical screening only but not isolation and structural elucidation of the responsible bioactive compounds. Ndukui et al. [69], for example, found saponins and steroid glycosides as the major secondary metabolites in *Ekebergia capensis* stem bark. Traces of tannins, anthraquinones, alkaloids, carotenoids, flavonoids, and anthracyanosides were also detected. Some of these secondary metabolites (tannins, phlobatannins, glycosides, phenols, saponins, quinones, terpenoids, and steroids) were also detected in *Plumbago zeylanica* [70]. It is worth noting that none of these studies probed into the mechanism of action of the extracts.

**Figure 6:**
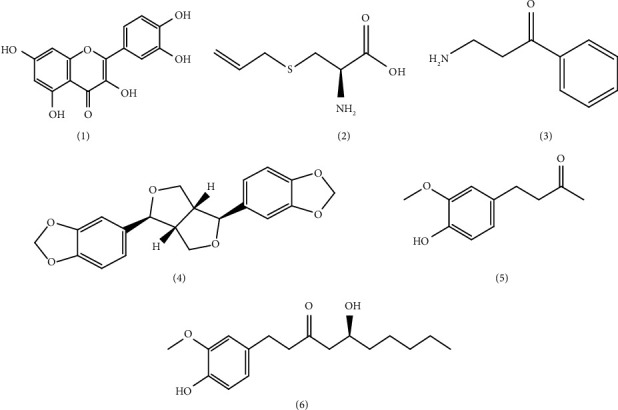
Some of the bioactive molecules characterized from extracts of plants reported in EAC for treatment of ED and infertility, and enhancing fertility and virility (based on studies outside the region). The numbers **1** to **6** refer to the molecules mentioned in Table 3.

We, therefore, performed further searches and retrieved other 9 species (along with *Zingiber officinale*) cited in the EAC that have been explored for their phytochemical profiles as well as aphrodisiac, procopulatory, and fertility effects (Table 3). One of the most studied plants in this context is *Allium cepa* (*A*. *cepa*) which is locally used in culinary recipes. It has been reported to improve copulatory behaviour in sexually experienced rats [71]. Malviya et al. [72] indicated that ethyl acetate fraction of *A*. *cepa* bulb at 200 mg/kg restored the mating behaviour (ejaculatory latency, postejaculatory interval, mount, intromission, and ejaculatory frequencies and mount and intromission latencies) of drug-mediated sexually dysfunctional male rats. Quercetin (**1**) (Figure 6), a flavonoid present in extracts of *A*. *cepa*, enhanced sperm motility through the regulation of protein kinase C-mediated activation of the human voltage-gated proton channel and could explain its therapeutic effect when used in the treatment of human infertility [14]. Similarly, S-allyl cysteine (**2**) isolated from *Allium sativum* restored erectile function in diabetic rats through inhibiting reactive oxygen species formation via modulation of nicotinamide adenine dinucleotide phosphate oxidase subunit expression in penile tissues [73].

The third highly investigated species is *Mondia whitei*. It has been found to increase sexual arousal and copulatory efficiency and improve sexual sensation in rats [74–78]. A follow-up study with a polyherbal formulation containing *Mondia whitei*, *Dracaena arborea*, and *Bridelia ferruginea* deduced that the administration of the formula enhanced the sexual performances and increased the mounting and intromission frequencies of normal rats and prediabetic rats [79].

*Zingiber officinale* (ginger) is the most thoroughly studied plant cited in this report. A bioactive compound from this species (zingerone, **5**) attenuated zearalenone-induced steroidogenesis impairment in TM3 Leydig cell lines [80] and elicited dose-dependent enhancement of fertility in male and female rats as witnessed by increments in gonadal weights and sperm counts [81]. A gingerol (**6**)-rich fraction of ginger at 50, 100, and 200 mg/kg when administered to male rats with carbendazim‐induced toxicity led to increased sperm motility and count but attenuated sperm abnormality [82].

Herbal extracts from plants such as *Allium cepa*, *Allium sativum*, *Mondia whitei*, and *Zingiber officinale* improve semen quality and sperm parameters such as concentration, viability, motility, morphology, and DNA integrity through increment in gonadal hormone levels (testosterone and luteinising hormone), sequestering free radicals and enhanced production of nitric oxide [83–85]. Such studies substantiate that the traditional claims of using the plants in the treatment of sexual dysfunction in EAC may be credible.

The contraceptive effect observed in plants such as *Catha edulis* (cathinone) and *Cannabis sativa* seed extracts is supported by studies which instead link their use to ED [86]. Nevertheless, plant extracts from certain families have been shown to elicit contradictory effects in fertility studies. Such differential bioactivities are species-specific and may depend on the extraction method and solvents employed [87, 88].

### 3.5. Clinical Studies

Clinical evaluation of herbal products is a requirement before they are promoted and used. In this study, we did not find any clinical studies in EAC that was performed on the extracts or isolated compounds from the cited plants. Further searches for global reports indicated that *Zingiber officinale* is the only plant encountered in this study that have been subjected to clinical studies investigating its effect on male ED, female sexual function, and infertility [89]. For example, its capsules improved the sexual function and quality of life in four weeks of a randomized, double-blind clinical trial involving women of reproductive age (*n* = 190) [90]. Another randomized double-blind placebo-controlled trial found that 3-month oral treatment using 500 mg/powder/day reduced sperm DNA fragmentation in infertile men [91]. Such promising clinical results demonstrate the need for more clinical trials on species such as *Mondia whitei*, *Acalypha villicaulis*, *Combretum illairii*, *Erythrina abyssinica*, *Pappea capensis*, *Rhus vulgaris*, and *Warburgia ugandensis* that are widely used in the region.

### 3.6. Adverse Side Effects and Toxicity of Medicinal Plants and Bioactive Phytochemicals Reported

Further analysis of reports considered in this systematic review showed that no ethnobotanical survey captured the side effects of herbal preparations used in the management of sexual dysfunction, infertility, and improving virility in the EAC. However, some of the plants such as *Abrus precatorius* (roots, leaves, and seeds) cited in the EAC are known to contain highly poisonous compounds (abrine, precatorine, and hypaphorine) [139]. It could be positioned that the preparation of remedies with more than one plant and plant part or with the addition of adjuvants may be a way of masking the toxicity of the medicinal plants [36, 93].

From available toxicological studies, extracts from six of the investigated bioactive species have been shown to be safe (Table 4). Four species (*Abrus precatorius*, *Catha edulis*, *Cannabis sativa*, and *Parquetina nigrescens*) have been indicated to elicit marked toxicity, indicating that their use may lead to adverse reactions in herbal medicine practice. For bioactive compounds identified in the listed species (Table 3), quercetin (**1**) is potentially cytotoxic and hepatotoxic at higher doses (100 to 2,000 mg/kg) [94, 95]. Similarly, cathinone (**3**) is a psychoactive compound that is toxic to sperm cells [96], and its abuse has been associated with fatal renal, hepatic, and cardiac injuries [97]. On the other hand, S-allyl cysteine (**2**) is considered to be safe, with very minor acute/subacute toxicity in mice and rats (LD_50_ > 54.7 mM/kg) when administered intraperitoneally [98]. Sesamine (**4**) is the major lignan in sesame seeds and has been confirmed to be safe. It attenuated reactive oxygen species and nitric oxide production in zebra fish (LD_50_) [99]. Toxicity studies with zingerone (**5**) and gingerol (**6**) have shown that they are safe [100, 104].

## 4. Conclusion

The EAC has a rich ethnobotanical knowledge of herbal remedies for the management of sexual dysfunction and infertility, and improving fertility and virility. Though we retrieved 171 medicinal plants being used, most of the species have not been subjected to phytochemical and bioactivity studies that lend credence to traditional claims of using them. We recommend performing toxicity studies and clinical trials using compounds isolated from some of the investigated species. Five highly cited unstudied species from this review (*Acalypha villicaulis*, *Combretum illairii*, *Erythrina abyssinica*, *Pappea capensis*, *Rhus vulgaris*, and *Warburgia ugandensis*) have been selected for further investigation of their phytochemistry, aphrodisiac, fertility, and phosphodiesterase-5 inhibitory activities.

## Figures and Tables

**Table 1:** Plants used in the management of sexual disorders, infertility, and improving sexual virility and fertility in the East African Community.

Plant family	Botanical name	Local name	Part used	Habit	Preparation mode	Administration	Use	Country	Reference
Acanthaceae	*Acanthus pubescens* (T. Thoms.) Engl.	Amatovu (Luganda)	Leaves	Shrub	Decoction	Oral in a local brew (*tonto*)	Aphrodisiac	Uganda	[61]

Acanthaceae	*Duosperma kilimandscharicum* (C. B. Clarke) Dayton	Not reported	Leaves, roots	Shrub	Decoction. Taken with goat blood or extract from goat meat	Oral	Aphrodisiac	Tanzania	[41]

Alliaceae	*Allium cepa* L	Katunguru (Runyankore)	Bulb, leaves, root tuber	Herb	Used directly, decoction	Oral in water, and in food or just chewed	ED	Uganda	[39, 65]

Alliaceae	*Allium sativum* L	Tungurusumu (Rukonjo)	Stem bulb, leaves, roots tuber	Herb	Used directly, decoction	Oral in water, and in food	ED	Uganda	[65]

Aloeaceae	*Aloe volkensii* Engl.	Hargeis, D'aar (Orma)	Leaves	Herb	Decoction	Used to wash genital area thrice daily	Infertility	Kenya	[58]

Anacardiaceae	*Mangifera indica* L	Muyembe (Luganda)	Bark	Tree	Decoction	Oral	Infertility (women)	Uganda	[22]

Anacardiaceae	*Ozoroa insignis* ssp. *reticulata* (Baker f.) J. B. Gillett	Not reported	Roots	Tree	Decoction	Oral	Aphrodisiac	Tanzania	[41]

Anacardiaceae	*Rhus vulgaris* Meikle	Mukanja (Runyankore), Mukanza (Rukonjo), Musatsa (Wanga)	Bark, roots, leaves, whole plant	Shrub	Used directly, decoction	Oral, chewed	ED, aphrodisiac	Uganda, Kenya	[41, 60, 65]

Annonaceae	*Ovariodendron anisatum*	Ndonga (Embu)	Whole plant	Herb	Decoction	Oral	ED, infertility	Kenya	[61, 62]

Annonaceae	*Uvaria acuminata* Oliv	Mundagoni, murori (Pokomo)	Roots	Shrub	Decoction. Used with *Markhamia zanzibarica*	Oral, a glass daily for 5 days	Aphrodisiac	Kenya	[59]

Annonaceae	*Uvaria leptocladon*	Sholole (Orma)	Roots	Shrub	Decoction. Used with *Boscia coriaceae* and *Combretum hereroense*. For infertility, use with *Croton dichagamus*	Oral, half a glass daily for 5 days. For infertility, half glass thrice daily for 3 days	ED, infertility (women)	Kenya	[58, 59]

Apocynaceae	*Carissa spinarum* L. (Synonym: *Carrisa edulis* Forssk. (Vahl))	Leketetwo (Marakwet), Logetetwa (Pokot), Omukuyomonza	Roots, bark	Shrub	Decoction with *Elaedendron* buchanannii bark or powder mixed with *Tragia furialis*	Oral, taken as tea	Aphrodisiac	Tanzania, Kenya	[64]

Apocynaceae	*Acokanthera schimperi* (A.D.C) Schweinf	Not reported	Roots	Tree	Infusion of powder	Oral	Aphrodisiac	Kenya	[65]

Apocynaceae	*Landolphia swynnertonii*	Mokokwet (Marakwet)	Roots	Shrub	Decoction	Oral	Increasing fertility (women)	Kenya	[66]

Apocynaceae	*Mondia whitei* (Hook. f.) Skeels	Omulondo (Luganda), Omurondo (Runyankore), Mukombelo (Luhya)	Roots/root bark	Vine	Used directly (chew when raw or dry), decoction	Oral in tea and in food	ED, aphrodisiac, fertility enhancer	Uganda, Kenya	[22, 23, 27, 39, 41, 65, 67–72]

Apocynaceae	*Cryptolepis obtusa* N. E. Br	Not reported	Roots	Shrub	Decoction (Tanzania), used directly	Drunk, chewed	Aphrodisiac	Kenya, Tanzania	[73]

Apocynaceae	*Dregea rubicunda* K. Schum	Not reported	Roots	Climber	Used directly	Fresh root chewed	Aphrodisiac	Tanzania	[60]

Apocynaceae	*Pachycarpus robusta* (A. Rich.) Bullock	Not reported	Roots	Herb	Not specified	Not specified	Aphrodisiac	East Africa	[41]

Apocynaceae	*Parquetina nigrescens* Afzel	Not reported	Roots	Climber	Decoction	Oral, once in the evening	Aphrodisiac	Kenya	[60]

Apocynaceae	*Periploca linearifolia* Dill. & Rich	Sinendet (Nandi)	Roots/milky latex	Climber/liana	Decoction	Oral	Fertility	Kenya	[75]

Arecaceae	*Phoenix reclinata* Jacq	Akakindo, Mukindo (Runyankore), Mukindu (Pokomo)	Roots, leaves	Shrub	Decoction	Oral, a glass daily for 3 days	ED, aphrodisiac	Uganda, Kenya	[59, 65]

Asparagaceae	*Chlorophytum comosum* (Thunb.) Jacques	Nalwebe (Lusoga)	Tuber	Herb	Not specified	Not specified	Infertility	Uganda	[31]

Asphodelaceae	*Aspidoglossum biflorum* E. Mey	Drege (Kiswahili)	Roots	Herb	Not specified	Not specified	Aphrodisiac	Tanzania	[41]

Asteraceae	*Bidens pilosa* L	Mucege, Enyabarashana (Runyankore)	Shoot, flowers	Herb	Decoction of young flowers as tea	Oral, 500 ml daily for 2 days for ED	ED, increase fertility	Kenya, Uganda	[27, 76]

Asteraceae	*Launaea cornuta* (Hochst. Ex Oliv. & Hiern) C. Jeffrey	Uthuunga (Kikamba)	Leaves, stems	Herb	Infusion	Oral	Infertility (women)	Kenya	[77]

Asteraceae	*Lactuca inermis* Forssk. (L. Capensis Thurnb.)	Not reported	Roots	Herb	Not specified	Not specified	Aphrodisiac	Uganda	[56]

Asteraceae	*Microglossa pyrifolia* (Lam.) Kuntze	Omuhe/Mkuraiju	Leaves	Herb	Not specified	Not specified	Aphrodisiac	Tanzania	[41]

Asteraceae	*Sonchus asper*	Iviuviu (Embu)	Whole plant	Herb	Decoction	Oral	ED	Kenya	[61]

Asteraceae	*Psiadia punctulata* (DC.) Vatke	Konocho (Marakwet), Shiro (Luhya)	Roots	Herb	Decoction	Oral	Aphrodisiac, sterility (men)	Kenya	[23]

Asteraceae	*Sonchus schweinfurthii* Oliv. & Hiern	Sungasunga	Roots	Herb	Decoction	Oral	Aphrodisiac	Tanzania	[78]

Asteraceae	*Tagetes minuta* L	Nyiramunukanabi (Kinyarwanda)	Shoot	Herb	Decoction	Oral	Increase fertility, ED	Kenya, Rwanda	[76, 79]

Asteraceae	*Vernonia cinerea* (L.) less (or *Cyanthillium cinereum* (L.) H. Rob.)	Kayayana (Luganda)	Leaves, roots	Shrub	Used directly, decoction	Oral	ED	Uganda	[65]

Asteraceae	*Vernonia lasiopus* O Hoffin	Shiroho	Roots	Shrub	Decoction/infusion	Oral, infusion drunk twice a day	Aphrodisiac	Kenya	[60]

Balanitaceae	*Balanites aegyptiaca* Del	Ng'oswet (Marakwet)	Roots	Tree	Decoction	Oral	Increasing fertility (women)	Kenya	[66]

Balsaminaceae	*Impetiens* species	Entungwa baishaija (Runyankore)	Whole plant	Herb	Used directly (chew), decoction	Oral	ED	Uganda	[39, 65]

Balsaminaceae	*Impatiens tinctoria* A. Rich	Chemakalbayi	Roots	Herb	Decoction	Oral	Fertility	Kenya	[80]

Bignoniaceae	*Markhamia zanzibarica*	Mubwoka (Pokomo)	Roots	Tree	Decoction. Used with *U*. *acuminata* roots. For infertility, it is used with *Salvadora persica* and *Uvaria acuminate*	Oral, a glass (or twice for infertility) daily for 5 days	Aphrodisiac (men), infertility (women)	Kenya	[58, 59]

Bignoniaceae	*Spathodea campanulata* Buch. -Harm. ex DC	Kifabakazi	Bark	Tree	Decoction	Oral	Infertility (men)	Uganda	[22]

Boraginaceae	*Ehretia cymosa* Thonn	Morori (Marakwet), Ponponat (Pokot), Shekhutu (Luhya)	Roots, leaves	Shrub	Decoction	Oral	Aphrodisiac	Kenya	[23, 75]

Boraginaceae	*Kigelia africana* Lam	Sausage tree (English)	Fruits, seeds	Tree	Decoction	Oral	Aphrodisiac	Tanzania	[81]

Canellaceae	*Warburgia ugandensis* Sprague	Mwiha (Runyaruguru), Mugeta (Embu)	Bark, leaves, roots	Tree	Decoction	Oral in tea, 1 spoonful thrice daily or in porridge; 250 ml drunk	ED	Uganda, Kenya	[27, 61, 65, 71]

Cannabaceae	*Cannabis sativa* L	Njayi, olusambya (Luganda), Njaga (Runyankore)	Leaves, roots	Shrub	Used directly (chew), decoction	Oral, inhaling fumes (smoking)	ED	Uganda	[65, 72]

Capparaceae	*Boscia coriaceae* Pax	Kalkacha (Orma)	Roots	Shrub	Decoction with *U*. *leptacladon* and *C*. *hereroense* roots	Oral, half a glass daily for 5 days	ED	Kenya	[59]

Capparaceae	*Boscia solicifolia* Oliv	Chelel (Marakwet)	Roots	Tree	Decoction	Oral	Increases fertility (male and female)	Kenya	[66]

Capparaceae	*Cadaba glandulosa* Forssk	Alakal (Orma)	Roots	Shrub	Decoction	Oral, half a glass daily for 5 days	Infertility (men and women)	Kenya	[58, 59]

Capparaceae	*Cadaba farinose*	Kumis (Orma)	Roots	Shrub	Decoction	Oral, half a glass daily for 3 days	Infertility (men and women)	Kenya	[58, 59]

Capparaceae	*Capparis tomentosa* Lam	Kumbolwop kimaget (Marakwet)	Roots	Climber	Decoction	Oral	Increasing fertility	Kenya	[66]

Capparaceae	*Cleome gynandra* L	Esobyo/Amarera (Runkonjo), Eshogi (Runyankore)	Leaves, roots, flowers	Herb	Used directly (chew), decoction	Oral or as food	ED, aphrodisiac	Uganda	[65, 82]

Capparaceae	*Cleome usambarica* Pax	Not reported	Roots	Herb	Infusion. Mixed with roots of *Macaranga usambarensis*	Oral, one cup is taken before food twice daily	Aphrodisiac	Kenya	[60]

Capparaceae	*Maerua triphylla* A. Rich	Chokotwa (Marakwet), Chokowa (Pokot), Olamalogi (Massai)	Stem bark, leaves	Tree	Infusion	Oral	Aphrodisiac	Kenya	[23, 28]

Celastraceae	*Catha edulis* Forsk	Mairungi (Runyankore)	Leaves, stem	Shrub	Used directly	Oral (chewed)	ED	Uganda	[39, 65]

Celastraceae	*Elaeodendron buchananii* Loes	Omuharanyi	Roots	Tree	Decoction or powder used in porridge	Oral	Aphrodisiac	Tanzania	[64]

Celastraceae	*Maytenus putterlickioides* (Loes.) Excell & Mendonca	Muthuthi	Roots	Shrub	Decoction	Oral	Aphrodisiac	Kenya	[60]

Celastraceae	*Maytenus senegalensis* (Lam.) Exell	Omuwaiswa (Lusoga)	Roots	Shrub	Not specified	Not specified	Infertility	Uganda	[31]

Celastraceae	*Pristimera andogensis* var. *volkensii* (Loes.) N. Hallé	Not reported	Roots	Climber	Infusion	Oral	Aphrodisiac	Kenya	[60]

Combretaceae	*Combretum constrictum* (Benth.) Laws	Not reported	Roots	Climber	Decoction with salt or used directly	Oral, a cup drunk twice a day; root chewed	Aphrodisiac	Kenya	[60]

Combretaceae	*Combretum hereroense* Schinz	Konkon (Orma)	Roots	Tree	Decoction. Used with *U*. *leptacladon*	Oral, a glass daily until effective	ED, infertility	Kenya	[59, 83]

Combretaceae	*Combretum illairii* Engl	Mshinda alume (Pokomo)	Root bark	Tree	Decoction. Used with *Grewia tenax* for men	Oral, a glass daily for 7 days (or 2-3 times daily for 14 days for infertility in women)	ED, infertility (men & women)	Kenya	[58, 59, 83]

Combretaceae	*Combretum molle* R. Br. ex G. Don	Omurama (Runyangkore)	Leaves	Tree	Decoction	Drink 500 ml (adult) daily	ED	Uganda	[27]

Combretaceae	*Combretum pentagonum* Laws	Not reported	Roots	Climber	Decoction with salt or used directly	Oral, a cup drunk twice/thrice a day or root is chewed	Aphrodisiac	Kenya	[60]

Cucurbitaceae	*Cucurbita maxima*	Ocwica (Lango)	Leaves/seeds	Herb	Decoction/used directly	Stew eaten or raw seeds chewed twice daily	Aphrodisiac	Uganda	[82]

Ebenaceae	*Flueggea virosa* (Roxb. Ex Willb.) Voigt	Lukandwa/mukandula	Leaves	Shrub	Decoction	Oral	Infertility in women	Uganda	[22, 65]

Euphorbiaceae	*Acalypha villicaulis* Hochst. ex A. Rich	Kaisokampanga (Lusoga)	Roots	Shrub	Infusion	Oral	ED, aphrodisiac	Uganda, Kenya	[31, 60, 65]

Euphorbiaceae	*Clutia abyssinica* Jaub & Spach	Kapkurelwo (Marakwet)	Roots	Shrub	Decoction	Oral	ED	Kenya	[84]

Euphorbiaceae	*Croton dichagamus*	Qashin a'adha, Muuqaadhi (Orma)	Roots	Tree	Decoction. Used with *Uvaria leptocladon* roots	Taken, half glass 3 times daily for 6 days	Infertility in women	Kenya	[58]

Euphorbiaceae	*Croton menyharthii* Pax	Mualikaji, Muyama (Pokomo)	Roots, leaves	Tree	Decoction	Oral, half glass 2-3 times daily for 5 days	Infertility in women	Kenya	[58]

Euphorbiaceae	*Erythrococca fischeri* Pax	Mboga (Pokot)	Roots	Shrub	Decoction	Oral	Infertility	Kenya	[23]

Euphorbiaceae	*Euphorbia candelabrum* Kotschy	Olpopongi	Roots	Tree	Not specified	Not specified	Infertility	Kenya	[70]

Euphorbiaceae	*Euphorbia tirucalli* L	Not reported	Juice	Tree	Not specified	Not specified	Aphrodisiac	Tanzania	[41]

Euphorbiaceae	*Flueggea virosa* (Willd.) Voigt	Omukarara (Runyaruguru), Omukalali (Rukonjo)	Leaves, roots	Shrub	Decoction	Oral	ED, infertility	Uganda	[22, 65]

Euphorbiaceae	*Ricinus communis* L	Omukaakale (Lusoga)	Leaves	Shrub	Not specified	Not specified	ED	Uganda	[31]

Euphorbiaceae	*Tragia benthamii* Baker	Kamyu (Luganda)	Roots	Herb	Decoction	Oral	ED	Uganda	[71]

Euphorbiaceae	*Tragia brevipes* Pax	Engyenyi (Runyankore)	Leaves	Herb	Decoction	Oral	ED	Uganda	[65]

Euphorbiaceae	*Tragia furialis* Boj	Mgonampili	Roots	Climber	Decoction. Mixed with *Elaeodendron buchanannii* or *Spathodea campanulata* and *Carisa spinarum*	Oral	Aphrodisiac	Tanzania	[64]

Fabaceae	*Abrus precatorius*	Mudanda, muturituri, Mudwadwa (Pokomo)	Roots, leaves, seeds	Shrub	Used directly. Seed powder taken or with seed extract or powder of *Indigofera cordifolia* or stem powder of *Tinospora cordifolia*; root also chewed	Oral	Aphrodisiac	Kenya, Tanzania	[59, 60]

Fabaceae	*Abrus schimperi* Hochst. Ex Benth	Not reported	Roots	Shrub	Decoction	Oral	Aphrodisiac	Tanzania	[41]

Fabaceae	*Acacia brevispica* Harms	Kiptare (marakwet), Kiptara (Pokot)	Roots	Tree	Decoction	Oral	Aphrodisiac	Kenya	[23]

Fabaceae	*Acacia abysinica* Hochst ex.Benith	Munyinya (Runyankore)	Bark	Tree	Decoction	Oral	ED	Uganda	[39]

Fabaceae	*Acacia drepanolobium* Harms ex Sjöstedt	Eluai (Massai)	Stem bark	Shrub	Not specified	Not specified	For fertility	Kenya	[28]

Fabaceae	*Acacia nilotica* (L.) Delile	Ngobgwa (Marakwet), Kopokwo (Pokot)	Leaves, bark, roots	Tree	Decoction	Oral	Aphrodisiac	Kenya	[23]

Fabaceae	*Acacia reficiens* subsp. Misera (Vatke) Brenan	Leina (Marakwet), Panyarit (Pokot), Olchurrai (Massai)	Root/stem bark	Tree	Decoction	Oral	Aphrodisiac	Kenya	[23, 67]

Fabaceae	*Acacia sieberiana* Scheele	Munyinya (Runyankore, Runyaruguru)	Bark	Tree	Decoction	Oral	ED	Uganda	[65]

Fabaceae	*Afzelia africana* Pers	Eiya (Lugbara)	Bark	Tree	Decoction	Oral	Aphrodisiac	Uganda	[85]

Fabaceae	*Albizia coriaria* Welw ex Oliver	Omusisa (Runyankore)	Leaves, stem	Tree	Decoction	Oral	Aphrodisiac	Uganda	[39]

Fabaceae	*Arachis hypogaea* L	Ebinyobwa (Runyankore), Binyebwa (Rukonjo)	Seeds	Herb	Used directly (eaten raw or roasted)	Oral	ED	Uganda	[27, 65, 72]

Fabaceae	*Caesalpinia volkensii* Harms	Mucuthi, Muvuthi (Embu), Mujuthi (Meru)	Roots	Shrub	Used directly (eaten raw or cooked), taken with palm wine	Oral	Aphrodisiac	Kenya	[62]

Fabaceae	*Cajanus cajan* (L.) Millsp	Entondiirwa (Runyankore)	Leaves	Shrub	Decoction	Oral, drink 250 ml	ED	Uganda	[27]

Fabaceae	*Cassia abbreviata*	Mubaraka wa guba (Pokomo)	Roots	Tree	Decoction with *Cissampelos mucronata* roots	Oral, a glass 3 times daily for 4 days	ED	Kenya	[59]

Fabaceae	*Cassia didymobotrya* Fresen	Mugabagaba (Runyankore), Mukyora (Runyaruguru), Mucora (Rukonjo)	Leaves, roots	Shrub	Used directly (chew), decoction	Oral	ED	Uganda	[65]

Fabaceae	*Cassia occidentalis* L	Mwitanzoka (Runyankore, Rukonjo)	Leaves, roots	Herb	Used directly (chew), decoction	Oral	ED	Uganda	[65]

Fabaceae	*Desmodium salicifolium* Poir. DC	Mkongorana	Leaves, roots	Shrub	Decoction. Mixed with *Elaeodendron buchananii* and *Tragia furialis*	Oral, a glass taken daily	Aphrodisiac	Tanzania	[64]

Fabaceae	*Dolichos compressus* Wilczec	Chebugaa	Roots	Herb	Decoction	Oral	Fertility	Kenya	[80]

Fabaceae	*Eriosema psoraleoides* G.Don. Lam	Orutandaigwa	Leaves, roots	Shrub	Decoction	Oral	Aphrodisiac	Tanzania	[64]

Fabaceae	*Entada abyssinica* Steud. ex A. Rich		Stem, bark	Tree	Not specified	Not specified	Infertility	Kenya	[60]

Fabaceae	*Erythrina abyssinica* Lam. Ex DC	Jjiirikiti (Luganda), Omutembe (Kuria), Muhuti (Kikuyu), Oloponi	Bark (stem bark), roots, stem	Tree	Decoction	Oral, eaten	Infertility (in women)	Kenya, Uganda	[22, 70, 86]

Fabaceae	*Dichrostachys cinereal* (L.) Wight & Arn	Muremanjojo (Runyankore)	Bark	Tree	Decoction	Oral	ED	Uganda	[39, 65]

Fabaceae	*Macrotyloma axillare* (E.Mey.) Verdc	Akaihabukuru (Runyaruguru)	Leaves, roots	Herb	Decoction	Oral	ED	Uganda	[65]

Fabaceae	*Mucuna pruirens* (L.) DC	Mukuna	Seeds	Herb	Decoction (tea)	Oral	Aphrodisiac	Uganda	[85]

Fabaceae	*Prosopis juliflora*	Mathenge	Root bark	Tree	Decoction. Used with *Zanthoxylum usamel* root bark	Oral, one teaspoonful daily for 5 days	Infertility in women	Kenya	[58]

Fabaceae	*Senegalia brevispica* (Harms) Seigler & Ebinger	Not reported	Roots, stem	Tree	Not specified	Not specified	Aphrodisiac	Kenya	[87]

Fabaceae	*Vachellia nilotica* (L.) P. J. H. Hurter & Mabb	Not reported	Stem bark, roots	Tree	Decoction	Oral	Aphrodisiac	Tanzania	[41, 60]

Fabaceae	*Vachellia sieberiana* (DC.) Kyal. & Boatwr var. *vermoesenii* (De Wild.) Keay & Brenan	Not reported	Roots	Tree	Decoction	Oral	Aphrodisiac	Tanzania	[41]

Fabaceae	*Vigna unguiculata*	Bojo (Lango)	Leaves	Herb	Decoction (stewed)	Oral	Aphrodisiac	Uganda	[82]

Flacourtiaceae	*Ocoba spinosa* Forssk	Ekalepulepu (Ateso)	Roots	Herb	Decoction	Oral	ED	Uganda	[88]

Flacourtiaceae	*Xylotheca tettensis* (Klotzsch) Gilg	Not reported	Roots	Shrub	Decoction or used directly	Oral, taken or chewed	Aphrodisiac	Kenya	[60]

Lamiaceae	*Becium obovatum* (E. Mey. Ex. Benth) N. E. Br	Not reported	Not specified	Herb	Decoction	Oral	Genital stimulant/depressant	Kenya	[89]

Lamiaceae	*Hoslundia opposita* Vahl	Simbaywa (Pokot), Shikuma (Luhya), Mtserere (Giriama)	Roots, leaves	Shrub	Decoction	Oral, a glass 2-3 times daily for 14 days	Aphrodisiac, infertility (men and women)	Kenya	[23, 58, 59]

Lamiaceae	*Ocimum suave* Wild	Omujaaja (Runyangkore)	Leaves	Shrub	Decoction with rock salt	Oral, drink 500 ml	ED	Uganda	[27]

Lamiaceae	*Plectranthus barbatus* Andrews	Papaha (Pokomo), Kan'gurwet (Markwet)	Roots	Shrub	Decoction. Used with *C*. *rotundifolia* for first 4 days	Oral, half glass daily for 30 days	ED	Kenya	[59]

Loganiaceae	*Buddleia polystachya* Fres	Chorwet	Roots	Herb	Decoction	Oral	Fertility	Kenya	[80]

Lythraceae	*Punica granatum* L	Mukungumanga (Embu, mbeere), Kukumanga (Meru)	Seeds	Shrub	Decoction	Oral	ED, infertility	Kenya	[61, 62]

Malvaceae	*Adansonia digitata* L	Muramba (Embu), Mbamburi (Swahili)	Bark	Tree	Decoction	Oral	ED	Kenya	[61]

Malvaceae	*Dombeya burgessiae* Gerrard ex Harv	Mukusa (Luhya)	Bark	Tree	Used directly (chewed)	Oral	Aphrodisiac	Kenya	[23]

Malvaceae	*Hibiscus fuscus* Garcke	Cheptelia (Marakwet), Pkapuyan (Pokot)	Roots	Herb	Used directly (chewed)	Oral	Aphrodisiac	Kenya	[23]

Malvaceae	*Sida tenuicarpa* Vollesen	Keyeyo (Rukonjo)	Leaves	Herb	Decoction	Oral	ED	Uganda	[65]

Meliaceae	*Ekebergia capensis* Sparrm	Cape ash (English)	Stem bark	Tree	Decoction	Oral	ED	Uganda	[69]

Melianthaceae	*Bersama abyssinica* Fresen	Kipset (Marakwet)	Roots, leaves, branches, bark	Tree	Decoction (roots), used directly (leaves, branches and bark)	Oral	Aphrodisiac, infertility (women and men)	Kenya	[23]

Melianthaceae	*Xylocarpus benadirensis* Mattei	Not reported	Unripe fruits	Tree	Used directly (exudate used)	Oral	Aphrodisiac	Tanzania	[41]

Menispermaceae	*Cissampelos micronata* A. Rich	Chovi, kivila kya mani (Pokomo), kashikiropaka (Giriama)	Roots	Herb	Decoction. Used with *C*. *abbreviate*	Oral, half glass daily for 3 days	Aphrodisiac, infertility, azoospermia	Kenya	[59]

Moraceae	*Artocarpus integer* (Thunb.) Merr	Fenensi (Runyangkore)	Seeds	Tree	Decoction	Oral, taken as tea	ED	Uganda	[27]

Moraceae	*Ficus natalensis* Hochst	Ekitooma (Runyangkore)	Roots, root bark	Tree	Decoction	Oral, drunk 250 ml daily or 100 ml thrice daily (fresh root bark)	ED	Uganda	[27]

Moraceae	*Morus mesozygia* Stapf	Not reported	Roots	Tree	Decoction in cow/goat milk	Oral	Aphrodisiac	Kenya	[60]

Moringaceae	*Moringa oleifera* Lam	Moringa (English)	Seeds, leaves	Tree	Decoction, teas, food condiment	Oral; seed powder as tea; eat leaves as sauce; drink 100 ml	ED	Uganda	[27]

Myricaceae	*Myrica salicifolia* Hochst. ex A.Rich	Mujeje (Runyankore)	Roots, bark	Shrub	Decoction	Oral	ED	Uganda	[65]

Myrtaceae	*Syzygium guineense* (Willd.) DC	Lamaiwo (Marakwet), Cheptimanwa (Pokot)	Leaves, bark	Tree	Used directly (sap used)	Oral	Aphrodisiac, infertility	Kenya	[23, 60]

Olacaceae	*Capparis sepiaria* var. caffra	Hamwalika (Pokomo), Mugwada paka (Giriama)	Root bark	Shrub	Decoction. used with *Grewia plagiophylla*	Oral, half glass daily for 10 days	Aphrodisiac	Kenya	[59]

Passifloraceae	*Adenia gummifera* (Harv.) Harms	Mujoka (Pokomo)	Roots/stem	Climber	Decoction	Oral, half glass daily for 3 days	Infertility in women	Kenya	[58]

Pedaliaceae	*Sesamum indicum* L	Not reported	Leaves	Herb	Not specified	Not specified	Aphrodisiac	Kenya	[41]

Phytolaccaceae	*Phytolacca dodecandra* L'Her	Muhoko (Runyankore), Ruhuko (Rukonjo)	Roots, leaves	Shrub	Used directly	Smear on ripe banana and roast	ED	Uganda	[65]

Piperaceae	*Piper umbellatum* L	Not reported	Roots	Climber	Decoction with *Aframomum* roots and strained	Oral, one cup taken daily	Aphrodisiac	Kenya	[60]

Plumbaginaceae	*Plumbago zeylanica* L	Not reported	Roots	Shrub	Decoction	Oral	ED	Uganda	[70]

Polygalaceae	*Polygala aphrodisiaca* Gürke	Not reported	Roots	Herb	Decoction, i.e., cooked with a young cock	Oral, eaten in food	Aphrodisiac	Tanzania	[41]

Polygalaceae	*Polygala sphenoptera* Fresen	Not reported	Roots	Herb	Infusion	Oral	Aphrodisiac	Kenya, Tanzania	[23, 41, 60]

Polygalaceae	*Securidaca longipedunculata* Fres	Omukondwa (Luganda)	Leaves, bark	Tree	Decoction	Oral	ED	Uganda	[72]

Polygonaceae	*Coffea* species	Mwani (Runyankore)	Seeds	Shrub	Roasted and chewed	Oral as a beverage	ED	Uganda	[65]

Polygonaceae	*Hallea rubrostipulata* (K. Schum.) J. F. Leroy	Muziiko (Runyankore)	Bark, roots	Tree	Decoction	Oral	ED	Uganda	[65]

Polygonaceae	*Rumex abyssinicus* Jacq	Mufumbagyesi (Runyankore), Kasekekambaju (Luganda)	Leaves, stem	Shrub	Used directly (chewed)	Oral	ED	Uganda	[65]

Polygonaceae	*Rumex usambarensis* (Dammer) Dammer	Kaseke kambajjo (Luganda)	Leaves	Herb	Decoction	Oral	Aphrodisiac	Uganda	[61]

Polygonaceae	*Tarenna graveolens* (S. Moore) Bremek	Munyamazi (Rukonjo, Runyaruguru)	Leaves, roots, bark	Shrub	Decoction	Oral	ED, aphrodisiac	Uganda, Kenya	[65, 92]

Pteridaceae	*Actinopteris semiflabellata* Pic. Serm	Mwii wa ivia (Kikamba)	Whole plant	Herb	Infusion	Oral	Infertility in women	Kenya	[77]

Ranunculaceae	*Clematis hirsuta* Guill. & Perr	Omunkaamba (Runyagkore)	Leaves	Vine	Decoction	Oral	ED	Uganda	[27]

Rhamnaceae	*Berchemia discolor* (Klotsch) Hemsl	Muchukwo (Marakwet)	Roots	Tree	Decoction	Oral	ED	Kenya	[84]

Rubiaceae	*Coffea arabica* L	Mwani (Runyankore)	Seeds	Shrub	Roasted and chewed	Oral as a beverage	ED	Uganda	[39, 65]

Rubiaceae	*Coffea canephora* Pierre ex A. Froehner (synonym: *Coffea robusta*)	Omwaani (Runyankore)	Leaves, fruits, seeds, roots	Shrub	Decoction of leaves and fruits, used directly (chew seeds) or cook with food	Oral	ED, aphrodisiac	Uganda, Kenya	[27, 60, 72]

Rubiaceae	*Craterispermum schweinfurthii* Hiern	Not reported	Roots	Shrub	Decoction, used directly (chew)	Oral	Aphrodisiac	Kenya	[60]

Rubiaceae	*Heinsia crinite*	Not reported	Stem bark	Shrub	Not specified	Not specified	ED	DRC	[93]

Rubiaceae	*Molinda citrifolia* Benth	Muziiko (Runyankore)	Roots	Tree	Decoction	Oral	ED	Uganda	[39]

Rubiaceae	*Psychotria capensis* subsp. *riparia* (K. Schum. & K. Krause) Verdc	Not reported	Roots	Shrub	Infusion/decoction	Oral	Aphrodisiac	Kenya	[60]

Rubiaceae	*Psychotria cyathicalyx* E.M. A. Petit	Not reported	Roots	Shrub	Decoction	Oral	Aphrodisiac	Kenya	[60]

Rubiaceae	*Psychotria lauracea* (K. Schum.) E. M. A. Petit	Not reported	Roots	Shrub	Decoction	Oral	Aphrodisiac	Kenya	[60]

Rubiaceae	*Vangueria infausta* Burch	Tabirirwo (Marakwet), Komolwo (Pokot)	Roots	Shrub	Decoction	Oral	ED, infertility	Kenya	[23]

Rutaceae	*Citropsis articulata* Swingle & Kellerman	Katimbolo (Luganda), Muboro (Runyankore)	Roots, bark	Tree	Decoction, used directly (chew)	Oral as a beverage in tea	ED, aphrodisiac	Uganda	[39, 61, 65]

Rutaceae	*Citrus sinensis* (L) Osbeck	Mudimu (Giriama)	Roots/stem bark	Tree	Decoction. Mixed with *Acacia robusta* and *Cissus rotundifolia* roots	Oral, a glass 3 times daily for 3 days	Infertility in women	Kenya	[58]

Rutaceae	*Fagaropsis hildebrandtii* (Engl.) Milne-Redh	Muvindavindi (Kamba)	Leaves	Shrub	Decoction	Oral	Infertility	Kenya	[94]

Salicaceae	*Flacourtia indica* (Burm.f.) Merr	Tungururwo (Marakwet), Tingoswa (Pokot)	Roots	Tree	Decoction	Oral	Infertility	Kenya	[23]

Sapindaceae	*Allophylus pervilleria* (A.Rich) Engl	Mnyanga kitswa (Pokomo)	Roots	Shrub	Decoction	Oral, a glass daily for 7 days	Infertility (men and women)	Kenya	[58, 59]

Sapindaceae	*Cardiospermum halicacabum* L	Akambula (Lusoga)	Leaves	Climber	Not specified	Not specified	Infertility	Uganda	[31]

Sapindaceae	*Pappea capensis* Eckl. & Zeyh. var. radlkoferi Schinz	Oltimigomi (Massai)	Bark	Tree	Decoction	Oral	Aphrodisiac	Kenya, Tanzania	[28, 41, 70]

Solanaceae	*Capsicum frutescens* L	Kamurari (Luganda), Eshenda (Runyankore)	Fruits, leaves, bark	Herb	Used directly (chew), decoction	Orally in food	ED	Uganda	[65, 72]

Solanaceae	*Solanum incanum* L	Labotwa (Marakwet), Lopotwo (Pokot), Maduranzura (Luhya)	Roots	Herb	Used directly (chew)	Oral	ED	Kenya	[23]

Solanaceae	*Solanum nigrum* L	Managu (Embu)	Whole plant	Herb	Decoction	Oral	ED	Kenya	[61]

Sterculiaceae	*Cola acuminata* Schott & Endl	Engongoli (Rukonjo, Runyaruguru)	Fruits	Tree	Roasted and chewed	Oral in tea, porridge, milk	ED	Uganda	[65]

Sterculiaceae	*Sterculia africana* (Lou.r) Fior	Ililwo (Marakwet)	Seeds	Tree	Used directly (chewed)	Oral	ED	Kenya	[84]

Stilbaceae	*Nuxia floribunda* Benth	Mngogo	Roots	Tree	Decoction	Oral	Aphrodisiac	Tanzania	[78]

Tiliaceae	*Grewia plagiophylla*. K. Schum	Mkoi (Pokomo)	Root bark	Shrub	Decoction. Used with *C*. *sepiaria*	Oral, a glass daily for 10 days	Aphrodisiac	Kenya	[59]

Tiliaceae	*Grewia similis* K. Schum	Mukarara (Runyaruguru)	Leaves, bark	Shrub	Decoction	Oral	ED	Uganda	[65]

Tiliaceae	*Grewia tenax* (forssk.) Fiori	Deeka (Orma), Mubavubavu, mukawa wa guba (Pokomo)	Root bark	Shrub	Decoction. Used with *Combretum illairii*	Oral, a glass daily for 7 days	ED, aphrodisiac, infertility	Kenya	[58, 59]

Urticaceae	*Urtica massaica* Mildbr	Engyenyi (Runyankore)	Whole plant, roots	Herb	Decoction	Oral	ED, aphrodisiac	Uganda, Kenya	[65, 76]

Verbenaceae	*Clerodendrum myricoides* (Hocst.) Vatke	Munjuga iria	Roots, root bark	Shrub	Decoction	Oral	Aphrodisiac	Kenya	[41, 76]

Vitaceae	*Cissus rotundifolia* (forsk.)	Mkwembe, Maneke, Neke (Pokomo), Arma (Orma)	Roots	Tree	Decoction. Sometimes used with *P*. *barbatus* for the first 4 days	Oral, a glass daily for 7 days	ED, increasing female fertility	Kenya, Tanzania	[59, 66]
	*Cyphostemma adenocaule* (Steud.ex. A. Rich) Desc.ex. Wild & R. B. Drumm	Akabombo akatono	Bark	Herb	Decoction	Oral	ED	Uganda	[71]

Zingiberaceae	*Zingiber officinale* Roscoe	Ntangahuzi (Runyankore)	Rhizome	Herb	Decoction	Oral in tea, milk, porridge	ED	Uganda	[65, 72]

Zygophylaceae	*Tribulus terrestris* L	Kilesan (Marakwet)	Whole plant	Herb	Used directly (chewed)	Oral	ED	Kenya	[84]

*Note*. ED: erectile dysfunction; languages: Luganda, Lusoga, Lango, Rukonjo, and Runyankore are spoken in Uganda; Marakwet, Luhya, Nandi, Kikamba, Pokot, Orma, Wanga, Pokomo, Massai, Giriama, and Swahili are spoken in Kenya and Kinyarwanda in Rwanda.

**Table 2:** Synopsis of the most used plant species for the treatment of infertility among men and women in the East African Community.

Medicinal plant	Parts used	Mode of preparation	Mode of administration	Group treated (country)	References
*Uvaria leptocladon*	Roots	Decoction	Oral, half glass thrice daily for 3 days	Women (Kenya)	[58, 59]
*Markhamia zanzibarica*	Roots	Decoction with roots of *Salvadora persica* and *Uvaria acuminata*	Oral	Women (Kenya)	[58, 59]
*Spathodea campanulata* Buch.-Harm. ex DC	Bark	Decoction	Oral	Men (Uganda)	[22]
*Mangifera indica* L	Bark	Decoction drunk	Oral	Women (Uganda)	[22]
*Flueggea virosa* (Roxb. Ex Willb.) Voigt	Leaves	Decoction drunk	Oral	Women (Uganda)	[22, 65]
*Erythrina abyssinica* Lam. Ex	Bark (stem bark), roots, stem	Decoction, eaten directly	Oral	Women (Uganda, Kenya)	[22, 70, 86]
*Cadaba glandulosa* Forssk	Roots	Decoction	Oral, half a glass daily for 5 days	Women and men (Kenya)	[58, 59]
*Cadaba farinose*	Roots	Decoction	Oral, half a glass daily for 3 days	Women and men (Kenya)	[58, 59]
*Combretum illairii* Engl	Root bark	Decoction used with *Grewia tenax* for men	Oral, a glass daily for 7 days (or 2-3 times daily for 14 days for infertility in women)	Women and men (Kenya)	[58, 59, 83]
*Hoslundia opposita* Vahl	Leaves	Decoction	Oral, a glass 2-3 times daily for 14 days	Women and men (Kenya)	[58, 59]
*Allophylus pervilleria* (A. Rich) Engl	Roots	Decoction	Oral, a glass daily for 7 days	Women and men (Kenya)	[58, 59]

**Table 3:** Bioactivity and phytochemical profile of some plants used in the treatment of ED and infertility, and enhancing virility and fertility in EAC.

Plant	Part used	Extract	Bioactivity/mechanism of action and active phytoconstituents
*Abrus precatorius*	Seeds	Methanol	Antifertility effect in rats [101].
*Allium cepa* L	Bulb	Aqueous, ethyl acetate	Enhanced copulatory behaviour in male rats [71, 106]. Ethyl acetate fraction of extract at 200 mg/kg restored the mating behaviour of drug-mediated sexually dysfunction male rats [72]. Quercetin (**1**) isolated from the plant-enhanced sperm motility [14].
*Allium sativum* L	Bulb	Aqueous, petroleum ether	Increased weight of seminal vesicles and epididymides in male rats [72, 106–108]. S-allyl cysteine (**2**) isolated from this species-promoted fertility [73].
*Cannabis sativa*	Seeds	Ethanol	Reduced epididymal sperm count in rats (contraceptive effect) [87, 88, 103].
*Catha edulis* Forsk	Shoots, small branches	Chloroform: diethyl ether extract (1 : 3)	Improvement of sexual behaviour and increase in plasma testosterone levels [112–114]. Cathinone (**3**) is toxic to sperm cells [96].
*Kigelia africana*	Fruits	Powder used directly	Increase sperm count, motility, and fertilization ability in African catfish increase in testicular weight, body weight, testosterone levels, and follicle-stimulating hormone [116, 117].
*Mondia whitei*	Root bark, roots	Aqueous, hexane	Increased sexual arousal, copulatory efficiency, sexual sensation [74–78] through the activation/stimulation of nitric oxide synthase activity resulting in the elevation of levels of cyclic guanosine monophosphate [83].
*Parquetina nigrescens*	Leaves	Aqueous	The extracts improved sexual activity, behaviour, and competence through improving sexual hormone secretion [102, 125].
*Sesamum indicum*	Seeds	Ethanol	It promotes body weight gain, seminal parameters, antioxidant action, and testosterone level [126]. Sesamin (**4**), a compound in this species, resisted cyclophosphamide-induced sperm nuclear maturity and DNA damage by increasing the expression levels of histones H2A and H2B in the testis [127].
*Zingiber officinale*	Rhizome		Powder at 100 mg has been cited to elicit positive effects in folliculogenesis and implantation [128]. Zingerone (**5**) isolated from the plant extract normalized zearalenone-impaired steroidogenesis in TM3 cells [80]. Similarly, a gingerol (**6**)-rich fraction of ginger enhanced sperm motility and count but attenuated sperm abnormality in male rats with carbendazim‐induced toxicity [82].

**Table 4:** Toxicity profile of plants with reports of efficacy that is used in the treatment of ED and infertility, and enhancing virility and fertility in EAC.

Plants	Toxicity reports
*Abrus precatorius*	Seeds contain abrin, a toxalbumin with a human lethal dose of 0.1–1 *μ*g/kg [148]. Poisoning is characterized by severe vomiting and abdominal pain, bloody diarrhoea, convulsions, and alteration of sensorium with depression of central nervous system [149].
*Allium cepa* L	Oral administration of extracts to mice at 250 and 500 mg/kg/day for 30–90 days had no visible toxicity symptoms. An oral dose of 30 g/kg/day for 30 days resulted into hypothermia, tachypnea, tachycardia, piloerection, and polyuria in the treated mice [150].
*Allium sativum* L	Its bulb extract induced mild alterations at 300 mg/kg in mice, indicating that it is relatively safe [151].
*Cannabis sativa*	Cannabidiol (a major nonpsychotropic constituent of this species) in extracts of this species is potentially toxic through the inhibition of hepatic drug metabolism, alterations of *in vitro* cell viability, reduced fertilization capacity, and decreased activities of *p*-glycoprotein and other drug transporters [152].
*Catha edulis* Forsk	Crude khat can damage the liver and kidneys and modulate levels of liver enzymes, urea, creatinine, and electrolytes essential for liver and kidney functions [153].
*Kigelia africana*	Low to moderately toxic [154].
*Mondia whitei*	Low toxicity in mice exposed to the extract for 90 days [155].
*Parquetina nigrescens*	Toxic to rats at 100 and 300 mg/kg of methanol leaf and aerial part extract. Renal haemorrhage, inflammation, and hepatic inflammation were noted [156].
*Sesamum indicum*	Ethanolic extract had low toxicity at 500 mg/kg body weight [157].
*Zingiber officinale*	Extract had no toxicity at 5,000 mg/kg body weight [158].

## Data Availability

This is a systematic review article, and no raw experimental data were collected. All data generated or analyzed during this study are included in this article.

## Supplementary Materials

Supplementary Materials Supplementary file 1: PRISMA 2020 checklist for the systematic review of medicinal plants used in the management of sexual dysfunction and infertility, and improving virility in the East African Community. Supplementary file 2: Risk of bias assessment of studies included for systematic review on medicinal plants used in the management of sexual dysfunction and infertility, and improving virility in the East African Community.
