# Supplementary material for: Medicinal Plants Used in the Management of Sexual Dysfunction, Infertility and Improving Virility in the East African Community: A Systematic Review
Source: Evid Based Complement Alternat Med. 2023 Aug 12;2023:6878852. doi: 10.1155/2023/6878852 (PMC10439835; doi:10.1155/2023/6878852)
Supplement: Supplementary Materials — Supplementary file 1: PRISMA 2020 checklist for the systematic review of medicinal plants used in the management of sexual dysfunction and infertility, and improving virility in the East African Community. Supplementary file 2: Risk of bias assessment of studies included for systematic review on medicinal plants used in the management of sexual dysfunction and infertility, and improving virility in the East African Community. [file 6878852.f1.zip › 6878852.f1/Supplementary File 2.pdf]

Supplementary file 2. Risk of bias assessment of studies included for systematic review on medicinal plants used in the management of sexual dysfunction, infertility and improving virility in the East African Community

S/N	Author name	Publication year	Q1	Q2	Q3	Q4	Total score	Risk of Bias
1	Watt and Breyer-Brandwijk	1962	1	0	0	0	1	Low
2	Kokwaro	1993	1	0	0	0	1	Low
3	Rwangabo and Rwangabo	1993	1	0	0	0	1	Low
4	Neuwinger	1996	1	1	0	0	2	Moderate
5	Munguti	1997	1	1	1	1	4	High
6	Kakudidi	2004	0	0	0	1	1	Low
7	Musila et al.	2004	1	0	1	0	2	Moderate
8	Kamatenesi-Mugisha and Oryem-Origa	2005	1	0	0	0	1	Low
9	Kareru et al.	2007	1	0	1	0	2	Moderate
10	Kitula	2007	0	0	0	0	0	Low
11	Ssegawa and Kasenene	2007	0	0	0	1	1	Low
12	Agea et al.	2008	1	0	0	0	1	Low
13	Jeruto et al.	2008	0	0	1	1	2	Moderate
14	Schmelzer	2008	1	1	1	1	4	High
15	Schmelzer and Gurib-Fakim	2008	1	0	1	0	2	Moderate
16	Kokwaro	2009	1	0	0	0	1	Low
17	Njoroge and Bussman	2009	1	0	0	0	0	Low
18	Okello et al.	2009	1	1	0	0	2	Moderate
19	Moshi et al.	2010	0	0	0	0	0	Low
20	Muriuki	2011	0	0	0	0	0	Low
21	Muthee et al.	2011	1	1	0	0	2	Moderate
22	Ndukui et al.	2012	1	1	1	1	4	High
23	Korir et al.	2012	1	1	1	1	4	High
24	Cheruiyot et al.	2013	1	0	0	1	2	Moderate

25	Kaingu et al.	2013a	1	0	1	0	2	Moderate
26	Kaingu et al.	2013b	1	0	1	0	2	Moderate
27	Okach et al.	2013	1	1	1	1	4	High
28	Kipkore et al.	2014	0	0	1	0	1	Low
29	Kimondo et al.	2015	0	0	0	1	1	Low
30	Kigenyi	2016	0	0	1	0	1	Low
31	Tugume et al.	2016	0	0	0	1	1	Low
32	Shiracko et al.	2016	1	1	0	1	3	Moderate
33	Ssozi et al.	2016	1	1	1	1	4	High
34	Wanzala et al.	2016	1	0	1	0	2	Moderate
35	Delbanco et al.	2017	1	0	1	0	2	Moderate
36	Kodi et al.	2017	0	1	1	1	3	Moderate
37	Tshibangu et al.	2017	1	1	1	1	4	High
38	Odongo et al.	2018	0	1	1	1	3	Moderate
39	Gumisiriza et al.	2019	0	0	0	1	1	Low
40	Nankaya et al.	2019	0	0	0	0	0	Low
41	Anywar et al.	2020	0	0	0	0	0	Low
42	Mbuni et al.	2020	0	0	0	0	0	Low
43	Asiimwe et al.	2021	0	0	1	1	2	Moderate
44	Nakaziba et al.	2021	0	0	0	0	0	Low
45	Onen et al.	2021	1	1	1	1	4	High
46	Kapere et al.	2022	1	0	0	0	1	Low

Q1 = Was the sample design appropriate for addressing the target population/study objectives?
Q2 = Were study participants sampled in an appropriate way?
Q3 = Was the sample size adequate?
Q4 = Were the study subjects and the setting described in detail that warrants the study undertaken?

Low (total score: 0 to 1), Moderate (total score: 2 or 3) or High (total score: 4)
